# Dietary diversity and associated factors among adolescent girls in Nifas Silk Laphto sub city, Addis Ababa, Ethiopia, 2021

**DOI:** 10.1186/s40795-023-00693-1

**Published:** 2023-03-07

**Authors:** Tarik Abebe, Getachew Sale Mezgebu, Fentaw Wassie Feleke, Meseret Tamrat

**Affiliations:** 1 Faculity of Public Health, Department of Nutrition and Dietetics, Jimma University, Jimma, Ethiopia; 2 School of Nutrition, Food Science and Technology, Hawassa, University, Hawassa, Ethiopia; 3 College of Medicine and Health Science, Woldia University, Woldia, Ethiopia

**Keywords:** Addis Ababa, Adolescent girls, Dietary diversity, Future generation, And malnutrition

## Abstract

**Background:**

Addressing the nutritional problems of adolescent girls is important as their nutritional status has a negative effect on the future generation. However, the evidence revealed the variation and unrelated data on the prevalence of dietary diversity and lack of including all adolescent age and community groups in Ethiopia. Hence, this study assessed dietary diversity and associated factors among adolescent girls in Nifas Silk Laphto Sub-city, Addis Ababa Ethiopia, 2021.

**Methods:**

A community-based cross-sectional study was conducted among 475 adolescent girls at Nifas Silk Laphto sub-city, Addis Ababa Ethiopia from July 1 to 30, 2021. Multistage cluster sampling was employed to select adolescent girls. Pretested questionnaires were used to collect the data. The data were checked for completeness and entered by Epidata version 3.1 and cleaned and analyzed by SPSS version 21.0. A multivariable binary logistic regression model was fitted to identify factors associated with dietary diversity scores. The degree of association was assessed using an odds ratio with a 95% confidence interval and variables with *p*-value ≤ 0.05 were considered significant.

**Result:**

The mean and the standard deviation of dietary diversity scores were 4.70 and 1.21 respectively. The proportion of low dietary diversity scores among adolescent girls was 77.2%. Adolescent girls’ age, meal frequency, wealth index of household, and food insecurity were significant determinants of dietary diversity score.

**Conclusion:**

The magnitude of low dietary diversity scores was significantly higher in the study area. Adolescent girls’, meal frequency, wealth index, and food security status were predictors of dietary diversity score. School-based nutrition education and counseling and designing strategies for improving household food security programs are crucial.

## Introduction

Adolescence is a transitional period marked by rapid and sequential physical and mental changes that transform a small child into a young adult girl. This age is a stage of growth and development in the lifespan that needs adequate and proper quality food to meet the nutrient requirement for their physical, mental growth and development in addition to reproductive maturity [1].

Dietary diversity is the consumption of an adequate variety of food groups [2]. A monotonous diet lacks essential micronutrients and contributes to the burden of malnutrition and micronutrient deficiencies [3]. The problem is particularly critical in adolescents because they need energy and nutrient-dense foods to grow and develop both physically and mentally and to live a healthy life [4].

Globally, only 17% of adolescents had got a diversified diet [5]. Similarly, in developing countries based on dietary diversity scores 23.5–50% of Iranian [6, 7] and 11.2% of Zimbabwe [8] adolescents got diversified diet. In Ethiopian findings from Jimma, south west of Ethiopia showed that 61.3% of adolescent girl students [9] and another study from Addis Ababa Yeka sub-city 43.3% of high school adolescent girls had low dietary diversity scores [10].

Most of the women in parts of sub-Saharan Africa, including Ethiopia, enter pregnancy with a poor nutritional status. It has been found that most of the time, the women may enter pregnancy with iron deficiency anemia and may have other micronutrient deficiencies which adversely affect her health and that of the fetus like low birth weight, neural tube defect and others [11–13].

Researches documented that maternal education [14], school type, mothers occupation, nutritional knowledge [15], residence and wealth status [14, 15] were associated with dietary diversity of adolescents. Eating behaviors of adolescents are influenced by many factors, including peer influences, parental modeling, food preferences, cost, personal and cultural beliefs, mass media, and body image perception [16, 17]. Mostly, household diets are predominantly starchy staples with few animal products and seasonal fruits and vegetables [9, 18].

Improving adolescent girls’ nutrition has benefits other than reproduction; the well-being and long-term nutritional health of women are legitimate goals in themselves [11]. In many low- and middle-income countries (LMICs) the double burden of malnutrition is high among adolescent girls, leading to poor health outcomes for the adolescent herself and sustained intergenerational effects [19]. In Ethiopia adolescent girl’s nutrition promotion is lagging and should connect with health services on one side, and food security programs on the other. Moreover, the evidence revealed the variation and unrelated data on the prevalence of dietary diversity and lack of including all adolescent age groups. Therefore, this study was assessed the dietary diversity practice and associated factors among adolescent girls in Nifas Silk Laphto sub city, Addis Ababa, Ethiopia, 2021.

## Methods

### Study area

The study was conducted at Nifas Silk Laphto sub city Addis Ababa city. Addis Ababa is the capital city of Ethiopia. The estimated population of the Addis Ababa city is 4.6 million. Nifas Silk Laphto sub city has 68.3 sq.km wide and total Population: 335,740, among them 158,126 are males and 177,614 female population. The sub-city also has 13 Woredas and 43,289 estimated household based on Nifas Silk sub city Data 2020 data [20].

### Study design and period

Community based cross-sectional study was conducted from July 1 to 30, 2021.

### Source and study population

All adolescent girls living in Nifas Silk Laphto sub-city, Addis Ababa Ethiopia were the source population. Meanwhile, randomly selected adolescent girls in Nifas Silk Laphto sub-city, Addis Ababa Ethiopia were study population. All adolescent girls living at least six months in randomly selected Ketenas at Nifas Silk Laphto sub-city, Addis Ababa Ethiopia included in the study. However, adolescent girls who were critically ill during the study and pregnant or lactating were excluded in the study.

### Sample size determination

The sample size was calculated for both prevalence and determinants of dietary diversity score. Finally, the maximum sample size was calculated by using the single population proportion formula: $n=\frac{{\left(Z\frac{\mathrm{\alpha }}{2}\right)}^{2}P(1-P)}{{d}^{2}}$; By considering the proportion of low dietary diversity score 0.75 among adolescent girl students a study done in the context of urban Northwest Ethiopia: 2017 [21], a confidence level of 95%: 1.96, 1.5 design effect and a 0.05 margin of error the sample size became 432. By adding 10% non-response rate, finally became 475.

### Sampling procedure

**Fig. 1:**
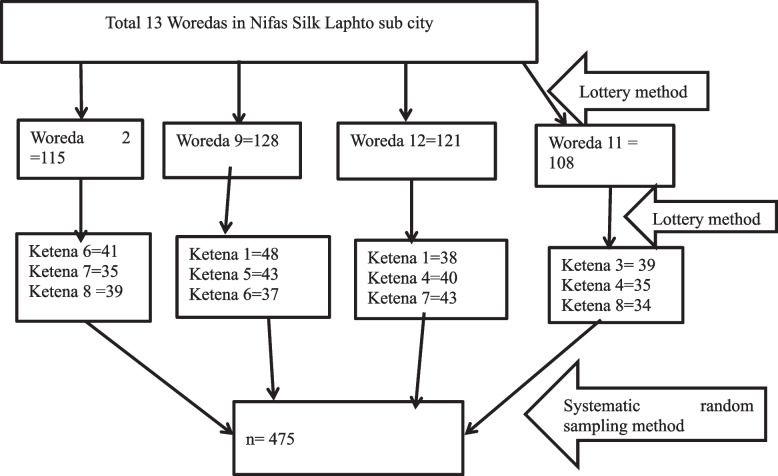
Schematic representation of sampling procedure

Multi-stage sampling technique was used to select the study participant. Adolescent girl from each household of selected Ketenas were identified using systematic sampling technique from Woredas youth center frame and home to home census survey until the required sample size fulfilled and the starting household were selected using a lottery method. If there were more than one adolescent per household, the study was conducted only from one of them by using lottery method (Fig. 1).

### Operational definitions

Dietary diversity score was considered as high, if the adolescents’ girl received greater than or equal to five food groups, which was created by summing up the number of food groups consumed over a 24-h period by an individual. Otherwise it is low dietary diversity score [9, 21, 22].

Household food security status was classified by HFIAS tool into two levels i.e. food secured if the household respondents responded ‘no’ to all of the items and insecure if the head of the household responded to at least one ‘yes’ to items of 1–9 [23].

Nutritional knowledge from twelve nutritional related questions responses: above mean value was considered as good and those below mean value was also considered as poor [9].

### Data collection tools and procedure

Data were collected by a semi-structured questionnaire developed through reviewing of literatures from different sources for socio-economic factors, dietary practice, comorbidity, food insecurity and nutritional knowledge [9, 14, 21, 24–26].

Dietary diversity and meal pattern were measured by dietary diversity questionnaire of 24 h dietary recall developed from FAO 2011 Guidelines for measuring household and individual dietary diversity of nine food group (i.e. starchy staples, cereals and white tubers), dark green leafy vegetables, other vitamin A-rich vegetables and fruits, other vegetables and fruits, organ meat, flesh meat, egg, legumes, nuts and seeds, milk and milk products,) were used to obtain information on subject’s food intake [27]. Subjects were asked to recall all foods eaten and beverages taken in the previous 24 h inside and outside the home. The DDS score was created by summing up the number of food groups consumed over a 24-h period by an individual from nine food groups.

Nutritional knowledge was assessed by 12 questions which aimed to assess whether adolescent girls and her mothers have had enough knowledge about the nutrients, advantage of diversified food and cause of malnutrition. A total nutrition knowledge score was obtained by adding the responses, scoring. A correct response was given a score of one, and an incorrect one had given a score of zero [9].

The Household Food Insecurity Access Scale were measured by the Household Food Insecurity Access Scale (HFIAS) measurement tool. Each of the questions asked with a recall period of four weeks. The respondents were first asked an occurrence question that is, whether the condition in the question happened at all in the past four weeks (yes or no) [23].

Wealth index also classified into tertile (poor, medium and rich) based on the EDHS 2016 list of household items by principal component Analyses [28].

### Data quality control

The data collectors were three nurse and two midwives and trained for one day regarding the purpose of the study and the procedures to be followed for data collection. The questionnaire was prepared in English first and then translated to Amharic and back to English by language experts to check for consistency. The semi-structured questionnaire was checked to avoid printing errors before data collection started. The name of the data collectors was recorded so as to enhance the responsibility to any incomplete data. Data collectors were summit the collected data to supervisor in daily basis and the supervisors will check the completeness of the data. Pre-test was done on 5% of the samples and through supervision during data collection.

### Data processing and analysis

The collected data were entered by using Epidata software version 3.1 and export, cleaned and analysed using SPSS version 21.0. Socio-demographic and other variables were presented by frequency tables and graphs. Binary logistic regression analysis was used to check association between dependent and independent variables. Bivariable binary logistic regression analysis was performed and variables with *p*-value < 0.25 in the bivariable analysis were exported to multivariate binary logistic regression analysis in order to screen strong predictors of dietary diversity score. The degree of association between dependent and independent variables was assessed using AOR at 95% CI. *P*-value less than 0.05 was considered as statistically significant. Multivariable binary logistic regression was performed using backward method. The adequacy of the model to predict the outcome variables was checked by Hosmer–Lemeshow goodness-of-fit and the *P-*value of 0.197 which was greater than 0.05 indicates the data were good fit to the model. Multicollinearity was checked by using variance inflation factor (maximum VIF = 1.83) of less than ten considered as there was no threat of multicollinearity.

## Result

### Socio-demographic characteristics of adolescent girls

A total of 460 adolescent girls were participated in the study yielding a response rate of 96.8%. The mean (± SD) age of the respondents was 14.55 (± 2.89) years and nearly more than half (51.1%) of the adolescent girls was in the age range of 10–14 years old. Among the total respondents 449 (97.6%) of them were single and half of (50.0%) attended at private school. Majority (58.0%) of adolescents were attended primary school and nearly all (92.0%) had media exposure like TV, radio, and other social Medias.

With respect to family related information’s, majority (51.7%) of their parents were married. Among the total respondents, nearly half (42.4%) of their mother educational status were college and above and nearly one third (28.0%) of mothers were government employee by occupation. More than one third (39.3%) of father educational status were college and above and 122(26.5%) were government employee. More than one third (38.4%) had poor wealth index and majority 265(57.6%) of households were food secured (Table 1).

**Table 1:** Socio-demographic characteristic of adolescent girls in Nifas Silk Laphto Sub city, Addis Ababa Ethiopia, 2021 (*n* = 460)

Variables	Category	Frequency	Percent
Age	Early	235	51.1
	Middle	92	20.0
	Late	133	28.9
Marital status	Single	449	97.6
	Married	11	2.4
Type of school attend	Government	230	50.0
	Private	230	50.0
Adolescent girl educational level	Primary school	267	58.0
	Secondary school	76	16.5
	Preparatory and above	117	25.4
Media exposure like TV, radio, and other social medias	Yes	423	92.0
	No	37	8.0
Marital status of parent	Married	238	51.7
	Single	60	13.0
	Divorced	91	19.8
	Widowed	71	15.5
Educational status mother	Unable to read and write	47	10.1
	Able to read and write	37	8.0
	Primary school (grade 1–8)	85	18.5
	Secondary school (grade 9–12)	96	20.9
	College and above	195	42.5
Occupation of mother	Housewife	102	22.2
	Government employee	129	28.0
	Private organization	87	18.9
	Merchant	95	20.7
	Daily laborer	47	10.2
Educational status of father	Unable to read and write	5	1.1
	Read and write only	28	6.2
	Primary school (Grade 1–8	103	22.5
	Secondary school (Grade 9–12)	143	31.1
	College and above	181	39.3
Occupation of father	Farmer	13	2.8
	Government employee	122	26.5
	Private organization	140	30.4
	Merchant	152	33.0
	Other^a^	33	7.1
Head of household	Father	232	50.4
	Mother	226	49.1
	Others	2	0.4
Who decide the type of food prepared in home	Father	363	78.9
	Mother	4	0.9
	Children	78	17.0
	Other	15	3.2
Family size	≤4	207	45.0
	> 4	253	55.0
Wealth index	Poor	177	38.4
	Medium	130	28.3
	High	153	33.3
Food security	Secure	265	57.6
	Insecure	195	42.3

^a^Others: daily laborer, students, retires

### Nutritional related knowledge and information’s

About half (50.9%) of the adolescent girls and 52.2% of their mothers had poor knowledge respectively. More than two-third (78.7%) of adolescent girls was obtained nutritional related information from schools and nearly all (85.2%) the respondents had not got any nutritional counseling from health and nutrition professionals. Few (5.9%) of respondents had history of chronic disease like Diabetes, kidney, heart disease etc. (Table 2).

**Table 2:** Nutritional Related knowledge among adolescent girls in Nifas Silk Laphto Sub city, Addis Ababa Ethiopia, 2021 (*n* = 460)

Variables	Category	Frequency	Percent
Adolescent girls nutritional related knowledge	Poor	234	50.9
	Good	226	49.1
Maternal nutritional related knowledge	Poor	240	52.2
	Good	220	47.8
Source of nutritional related information	Mass media	64	13.9
	Friends	2	.4
	Family	32	7.0
	School	362	78.7
Nutritional counseling from health professionals	Yes	68	14.8
	No	392	85.2
History of chronic disease like Diabetes mellitus, kidney etc	Yes	27	5.9
	No	433	94.1

### Dietary practice of adolescent girls

#### Meal pattern of adolescent girls

With respect to meal frequency majority (56.7%) of adolescent girls were more than three times per day. Regarding to snaking about 135(29.3%) of respondents had not any snaking habit and more than two-third (79.8%) skipping their breakfast ≤ 2 times per week. Majority (59.6%) of adolescent girls had eating out habit at least one times per week (Table 3).

**Table 3:** Meal pattern of adolescent girls in Nifas Silk Laphto Sub city, Addis Ababa Ethiopia, 2021 (*n* = 460)

Variables	Category	Frequency	Percent
Meal frequency	≤3	199	43.3
	> 3	261	56.7
Snaking	No	135	29.3
	Yes	325	70.7
Breakfast skipping	≤2 times per week	367	79.8
	> 2 times per week	93	20.2
Eating out/week	No	186	40.4
	Yes	274	59.6
Who influences your decision of meal	Parents	68	14.8
	Elder sibling	11	2.4
	Friends/class mate	152	33.0
	No one (I decide on my own)	229	49.8

#### Dietary diversity Score

**Fig. 2:**
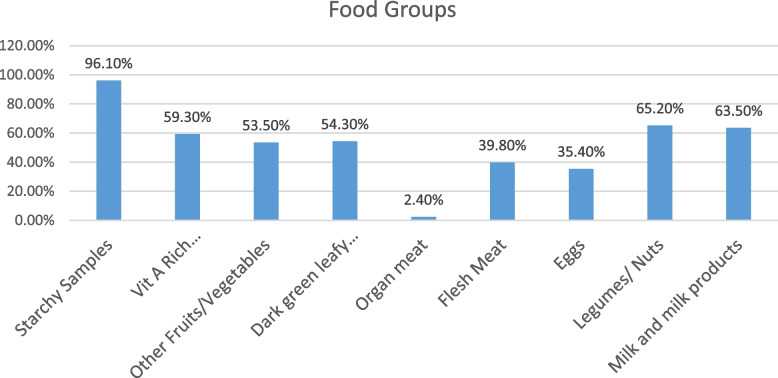
Types of food items consumed by adolescent girls in Nifas Silk Laphto sub city, Addis Ababa Ethiopia, 2021 (*n* = 460)

The mean dietary diversity score of study participants was 4.70 (SD: ± 1.21 respectively. The prevalence of low dietary diversity score among adolescent girls were 77.2% (95% CI: 73.3, 81.1). The majority 96.1% and 65.2% of adolescent girls consumed starch staples and Legumes/ Nuts respectively (Fig. 2).

#### Factors associated with dietary diversity score

Bivariable binary logistic regression analysis was done to assess association between individual independent variables and DDS to identify candidate variables for multivariable binary logistic regression. Age of adolescent girls, grade of adolescent girls, family size, meal frequency, adolescent girls and maternal nutritional related knowledge, marital status of parents, nutritional counseling, chronic disease, food security status and wealth index were significantly associated with (*P*-values < 0.25) and entered into multivariable binary logistic regression. Finally, adolescent girls age, meal frequency, wealth index, food security status were a statically significant predictors of dietary diversity score.

Adolescent girl found in the early age group were 4.19 times higher odds of low dietary diversity score than late adolescents [AOR, 4.19 (95% CI: 2.29, 7.66)]. Participants who had more than three meals per day were five times more likely to have low DDS than adolescent girls with meal frequency ≤ 3 times meal per day [AOR, 4.54 (95% CI: 2.29, 9.00)]. Adolescent girls living in the medium wealth index household were 73% less likely to have low DDS compared with those in rich wealth index [AOR, 0.27 (95% CI: 0.12, 0.57)]. The finding showed that participants who were in food secure households were 70% less likely having low DDS [AOR, 0.30 (95% CI: 0.16, 0.55)] (Table 4).

**Table 4:** Results of bivariable and multivariable binary logistic analysis of factors associated with dietary diversity score among adolescent girls in Nifas Silk Laphto sub city, Addis Ababa Ethiopia. 2021 (*n* = 460)

Predicators		DDS		COR (95%CI)	*P*-value	AOR (95%CI)	*P*-value
		**Low** **No (%)**	**High** **No (%)**				
Age	Early	211	24	6.01(3.48–10.4)	< 0.001	4.19(2.29–7.66)	< 0.001**
	Middle	65	27	1.64(0.93–2.90)	0.085	1.55(0.81–2.95)	0.143
	Late	79	54	1		1	
Grade	Primary	231	36	4.16(2.49–6.98)	< 0.001	1.01(0.24–4.27)	0.992
	Secondary	53	23	1.49(0.81–2.76)	0.201	1.04(0.37–2.79)	0.978
	Preparatory and above	71	46	1		1	
Parents marital status	Married	172	66	0.56(0.36–0.87)	0.010	0.75(0.43–1.31)	0.312
	Other	183	39	1		1	
Family size	≤4	173	34	1.99(1.26–3.14)	0.003	1.08(0.56–209)	0.613
	> 4	182	71	1		1	
Wealth index	Poor	133	44	0.30(0.16–0.58)	< 0.001	0.77(0.36–1.72)	0.485
	Medium	83	47	0.18(0.10–0.34)	< 0.001	0.27(0.12–0.57)	< 0.001**
	Rich	139	14	1		1	
Food security status	Food secure	178	87	0.21(0.12–0.35)	< 0.001	0.30(0.16–0.55)	0.001*
	Food insecure	177	18	1		1	
Adolescent girls nutritional knowledge	Poor	209	25	4.58(2.79–7.53)	< 0.001	0.71(0.25–2.03)	0.523
	Good	146	80	1		1	
Maternal nutritional knowledge	Poor	201	39	2.21(1.41–3.46)	0.001	1.56(0.91–2.65)	0.103
	Good	154	66	1		1	
Meal frequency	≤3 times	186	13	7.79(4.20–14.4)	< 0.001	4.54(2.29–9.00)	< 0.001**
	> 3 times	169	92	1		1	
Nutritional counseling	Yes	39	29	0.32(0.19–0.56)	0.002	0.63(0.34–1.17)	0.141
	No	316	76	1		1	
Chronic disease	Yes	16	11	0.41(0.18–0.89)	0.026	0.69(0.26–1.81)	0.450
	No	339	94	1		1	

*AOR* Adjusted odds ratio, *COR* Crude odds ratio

^*^Significant at P value < *0.05*

^**^ significant at p value < *0.001*

## Discussion

The findings of this study demonstrated that average dietary diversity score was 4.70(SD: ± 1.21) and the prevalence of low dietary diversity among adolescent girls was 77.2%. Adolescent girls age, meal frequency, wealth index, food security status were predictors of dietary diversity score.

The mean DDS was in line with a study in Jimma Town, South West Ethiopia which was 4.34(SD: ± 1.42) [9] and 4.69(SD: ± 1.46) in Ethiopian Gurage zone [14]. This result slight variation might occur because of the reference period difference to calculate DDS, the number of food groups included in the score, the study setting and lack of accessibility of diversified diet in the city.

The proportion of low dietary diversity was in line with a study done Gurage zone 73.2% [14], Gondar adolescent 75.4% [21] and higher when compared to another study done in Jimma town 12% of school adolescents had low [25], Dembia, northwest Ethiopia 32.3% [29], Addis Ababa, Yeka Sub-city 43.3% [10], Woldia 49.1% [30], South West Ethiopia 61.3% [9], and Iranian 50–76.5% [6, 7]. The difference might be due to variations of geographical location, seasonal variability, and other socio-demographic factors. Furthermore, the disparity might happen due to socio-economic differences and the presence of food-based dietary guidelines for other countries like Iran which promote diversified food consumption. However, this finding was lower when compared to a study done in Zimbabwe 88.8% [31] and global level which was only 17% of adolescents got diversified diet [5] . The disparity might happen due to socio-economic differences of the study area, production, availability and cultural preference.

Nearly all (96.1%) of adolescent girls consumed starchy staples. This finding was consistent with a study done in Gondar, Northwest Ethiopia: 97.7% of adolescent girls consumed starchy staples [21]. Another study in Jimma town also supports the current study [9]. This is because cereals were produced in the majority areas and highly accessible in the market, and the dietary habit of developing nations is entirely depends on starchy staples [32].

Those adolescent girls who lived in the medium wealth index household were 73% less likely to have low DDS compared with those in rich wealth index. This finding is inconsistent with studies conducted in Ethiopia Gurage zone and Jimma town [9, 14] and also in Dembia, northwest Ethiopia: inadequate dietary diversity was significantly associated with middle and high wealth category [29]. Although access is important, but the awareness of food-based dietary guidelines will probably have more effect. This might be due to variations of geographical location, seasonal variability, and market accessibility.

The finding also showed that participants who were in food secure households were 70% less likely having low DDS. This result is consistent with other findings done in Gondar and Nigeria reveals there is a significant positive relationship as expected between food security level and dietary diversity [33, 34]. Food insecurity has been shown to reduce individual-level consumption of ASF, fruits, and vegetables largely due to a significantly lower total food expenditure than food secure households [35, 36]. This implies that as the food security status improved and dietary diversity will be increased. Furthermore, DDS is measure of food security, nutrition information, early warning system and target of intervention at Global or national level [27, 37].

Adolescent girls found in the early age group were four times more likely to have low dietary diversity score than late adolescents. In fact, as the age increase, the educational status also increasing and this in turn improved the knowledge of diversified diet of adolescent girls, they have a chance to get information on healthy dietary habit. This difference also might be due to an exposure to different nutrition-related health education in the current study area [25].

Participants who had more than three meals per day were five times more likely to have low DDS than adolescent girls with meal frequency ≤ 3 times meal per day. The result is in line with a study done in West Java [38]. This might be due to the fact that as the number of meals increased per day the probability of getting a diversified diet will be rise.

### Strength and limitation of the study

The findings of this community-based study have a significant contribution to address the nutritional problems of adolescent girls. However, the cross-sectional nature of this study limits us from determining causal effects as the study variables. The study assessed household and individual dietary diversity only for the last 24 h; hence, there might be lack of a correct reflection of the usual dietary habits of adolescents and also leads to social disability bias.

## Conclusion

The magnitude of inadequate dietary diversity score was higher in the study area. Adolescent girl age group, meal frequency, wealth index, food security status were predictors of dietary diversity score. The federal ministry of health should focus on strengthening micro-finance and small business enterprise to increase access to food via amplified income, design strategies on household food security program [e.g., Productive Safety Net Program (PSNP)]. Moreover, starting nutritional education and counseling at all age and grade level are crucial. A large scale community based study with large sample size and more strong study design should be conducted.

## Data Availability

The datasets used and/or analyzed during the current study are available from the corresponding author.

## Abbreviations

AOR: Adjusted Odds Ratio
CDC: Centers for Disease Control and Prevention
CI: Confidence Interval
DDS: Dietary Diversity Score
DGLV: Dark Green Leafy Vegetables
FAO: Food and Agriculture Organization
IDA: Iron Deficiency Anemia
LIC: Low-Income Country
NGOs: Non-Governmental Organizations
PCA: Principal Component Analysis
SD: Standard Deviation
SPSS: Statistical Package for Social Sciences
WHO: World Health Organization
